# Poorer glycaemic control is associated with increased skin thickness at injection sites in children with type 1 diabetes

**DOI:** 10.1186/1687-9856-2014-2

**Published:** 2014-02-27

**Authors:** José G B Derraik, Marius Rademaker, Wayne S Cutfield, Jane M Peart, Craig Jefferies, Paul L Hofman

**Affiliations:** 1 Liggins Institute, University of Auckland, Auckland, New Zealand; 2 Dermatology Department, Waikato Hospital, Hamilton, New Zealand; 3 Gravida: National Centre for Growth and Development, Auckland, New Zealand; 4 Auckland Radiology Group, Auckland, New Zealand; 5 Starship Children’s Hospital, Auckland District Health Board, Auckland, New Zealand

**Keywords:** Type 1 diabetes, Dermis, Subcutaneous tissue, Subcutis, HbA1c

## Abstract

We aimed to assess the association between skin thickness and glycaemic control in children with type 1 diabetes. Forty-five children (51% males) aged 10.5 ± 2.1 years were studied. Thickness of skin layers were determined by ultrasonography, with participants having ultrasound scans of three anatomical regions (abdomen, thigh, and buttocks). Poorer glycaemic control (increasing HbA1c values) was associated with greater thickness of the dermis (p = 0.015), with an estimated thickening of 87 μm with every 1% increase in HbA1c. Our data suggest that dermal changes associated with poorer glycaemic control in adults are also observed in childhood.

## Correspondence/findings

### Introduction

Children and adults with type 1 diabetes mellitus have been shown to have thicker epidermis-dermis layer than controls [1,2], but these observations are not always consistent [3]. Previous studies suggested that neither the presence of abnormal plantar fascia thickness in diabetic adolescents [4] nor sclerodactyly in diabetic adults [5] was associated with glycosylated haemoglobin (HbA1c) levels. However, higher HbA1c levels (i.e. poorer glycaemic control) were associated with increased dermal thickness in adults [2]. We aimed to assess whether a similar relationship between skin thickness and glycaemic control occurs in children with type 1 diabetes.

### Methods

Otherwise healthy children and adolescents aged 5–14 years with type 1 diabetes mellitus were recruited from the diabetes clinics at Starship Children’s Hospital, Auckland, New Zealand [6]. Exclusion criteria included moderate to severe lipohypertrophy, other medical conditions such as coeliac disease or autoimmune thyroid disease, associated syndromes (e.g. Down’s syndrome) and other causes of diabetes (e.g. cystic fibrosis) [6]. Children with mild lipohypertrophy who were studied had no measurements made in areas where adipose thickening was noted.

Dermis and subcutis thicknesses were assessed using ultrasound at five common injection sites: anterior abdomen 3–4 cm lateral to the umbilicus (left- and right-hand sides), lateral mid-thigh (left and right thighs), and left buttock. From a clinical perspective it is most useful to understand skin thickness where insulin injections are administered, as skin thickness may ultimately influence the choice of needle length.

Dermal thickness was defined as the distance between the air-skin surface interface and the proximal aspect of the subcutaneous tissue layer, and included the small contribution of the epidermis. Subcutis thickness was measured from the proximal subcutaneous fat boundary to the underlying muscle fascia. Ultrasound was performed using an ATL HDI 5000 ultrasound machine (Phillips Healthcare, Best, Netherlands) and a 12 MHz linear array transducer. The exact site of needle insertion was marked prior to injection, and the transducer centered over this point. A single measurement was obtained mid-transducer, with cursors centered at the air-skin interface, the skin-subcutaneous fat interface, and the fat-muscle fascia interface. All ultrasound measurements were carried out by the same radiologist [JMP]. Note that a standoff was used to optimize image quality by increasing the distance between the transducer and the skin. This method of assessing depth of skin layers has been well-validated previously [7].

Apart from simple correlations, random effect mixed models with repeated measures (SAS v.9.3, SAS Institute Inc. Cary, NC, USA) were used to assess the association of HbA1c with skin thickness. Models included age, anatomical region, side (left or right), sex, and body mass index standard deviation score (BMISDS) as confounding factors. Descriptive data are presented as means ± standard deviations.

Ethics approval was provided by the Auckland District Health Board Research Review Committee. Written informed consent was obtained from parents/guardians, and verbal or written consent from each child as appropriate to their age.

### Results

Forty-five children (51% males) with type 1 diabetes were studied. Participants were aged 10.5 ± 2.1 years (range: 6.4–14.3 years) and of BMISDS 0.53 ± 1.01 (range: -1.61–2.99). Mean HbA1c was 8.0 ± 1.1% (64 ± 12 mmol/mol), with a range of 5.8–11.4% (40–101 mmol/mol). Higher HbA1c values (poorer glycaemic control) were correlated with increasing dermis thickness in buttocks (r = 0.35; p = 0.017), abdomen (r = 0.42; p = 0.004), and thigh (r = 0.44; p = 0.002). HbA1c values were also positively correlated with subcutis thickness in the abdomen (r = 0.37; p = 0.012), but not in buttocks (p = 0.10) or thigh (p = 0.45).

**Figure 1:**
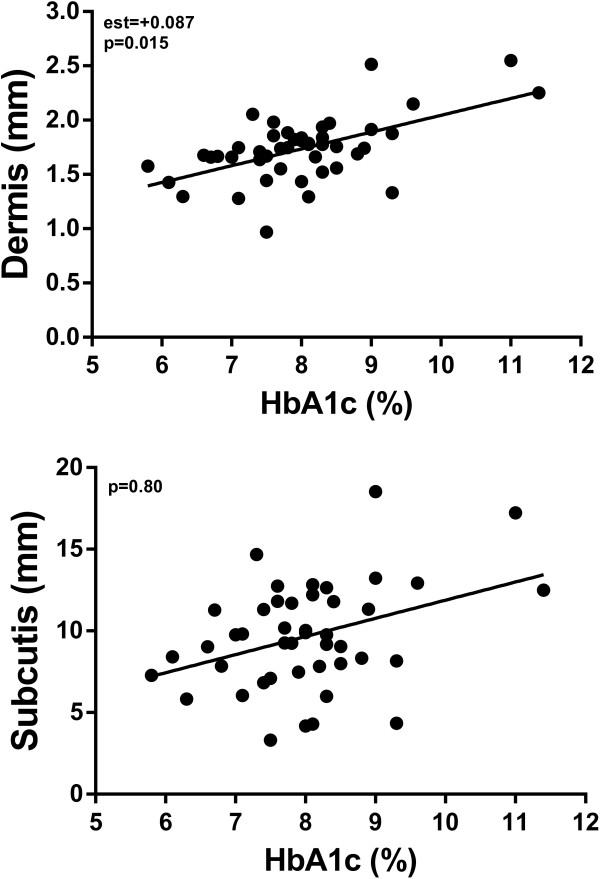
**The association between HbA1c levels and skin thickness in diabetic children (n = 45), as predicted by multivariate models.** For the sake of clarity, only the mean value of five samples for each individual (adjusted for confounding factors in multivariate models) was plotted.

Multivariate analyses showed that poorer glycaemic control (increasing HbA1c values) was associated with greater thickness of the dermis (p = 0.015), with an estimated thickening of 87 μm with every 1% increase in HbA1c (Figure 1). This association was observed despite adjustment for a number of confounders, including BMISDS that was highly associated with dermal thickness (p < 0.0001). There was no association between glycaemic control and subcutis thickness (p = 0.80).

### Conclusions

Our study suggests that changes in the dermis associated with poorer glycaemic control in adults [2] are also observed in childhood. Our findings are in contrast to those of Lo Presti *et al.*, who observed no association between HbA1c values and skin thickness or skin plus subcutis thickness [8]. The observed effect in our cohort might have resulted from the higher mean HbA1c (7.99 ± 1.09 vs 7.48 ± 0.77%; p = 0.011) and a wider range of HbA1c values in our patients compared to that previous study.

The relatively small number of participants in our study (n = 45) is a limitation. However, we obtained five measurements per child over three anatomical regions to account for between-site variations, and all measurement were performed by the same radiologist; thus, our data are likely to be robust.

An asymptomatic but measureable thickening of the skin commonly occurs in association with diabetes mellitus [9]. The clinical relevance of the dermal thickening observed in our study is unclear, but this thickening is one of a number of alterations in connective tissue that are observed in diabetic patients [2]. Although the mild skin thickening observed in our patients may not be clinically relevant, Buckingham *et al.* suggested that changes akin to scleroderma (i.e. greater thickening) may reflect generalized alterations in connective tissue in patients with diabetes, and possibly indicate increased risk of microvascular complications [10].

## Competing interests

The authors have no financial or non-financial conflicts of interest to disclose that may be relevant to this work. The funders had no role in study design, data collection and analysis, decision to publish, or preparation of this manuscript.

## Authors’ contributions

PLH, WSC, and CJD were responsible for the design of the research; JMP carried out the research; JGBD carried out the statistical analyses; JGBD and MR wrote the manuscript, with input from other authors. All authors read and approved the final manuscript.
